# Unraveling the complex metabolic nature of astrocytes

**DOI:** 10.3389/fncel.2013.00179

**Published:** 2013-10-11

**Authors:** Anne-Karine Bouzier-Sore, Luc Pellerin

**Affiliations:** ^1^Centre de Résonance Magnétique des Systèmes Biologiques, UMR 5536 CNRS/Université Bordeaux SegalenBordeaux, France; ^2^Laboratoire de Neuroénergétique, Département de Physiologie, Université de LausanneLausanne, Switzerland

**Keywords:** astrocytes, energy metabolism, aerobic glycolysis, lactate, glycogen, glucose

## Abstract

Since the initial description of astrocytes by neuroanatomists of the nineteenth century, a critical metabolic role for these cells has been suggested in the central nervous system. Nonetheless, it took several technological and conceptual advances over many years before we could start to understand how they fulfill such a role. One of the important and early recognized metabolic function of astrocytes concerns the reuptake and recycling of the neurotransmitter glutamate. But the description of this initial property will be followed by several others including an implication in the supply of energetic substrates to neurons. Indeed, despite the fact that like most eukaryotic non-proliferative cells, astrocytes rely on oxidative metabolism for energy production, they exhibit a prominent aerobic glycolysis capacity. Moreover, this unusual metabolic feature was found to be modulated by glutamatergic activity constituting the initial step of the neurometabolic coupling mechanism. Several approaches, including biochemical measurements in cultured cells, genetic screening, dynamic cell imaging, nuclear magnetic resonance spectroscopy and mathematical modeling, have provided further insights into the intrinsic characteristics giving rise to these key features of astrocytes. This review will provide an account of the different results obtained over several decades that contributed to unravel the complex metabolic nature of astrocytes that make this cell type unique.

As often in physiology, the role(s) of specific cell types is(are) suggested initially by their morphology, localization and interactions with other elements in the tissue where they are found. This was made possible by the development of microscopy and various histological techniques. Astrocytes are no exception and it is quite instructive to recollect the historical descriptions (by those who made them) that led to the hypothesis of an important metabolic role of astrocytes in the central nervous system. For a more extensive historical perspective about the emergence of the concept of Neuroglia, the reader is referred to Somjen (1988) or Kettenmann and Verkhratsky (2008).

## A STAR IS BORN

The first description of a distinct tissue from neurons was attributed to the german anatomist Rudolf Virchow as he named it “nervenkitt” or “neuroglia” (Virchow, 1856) to reflect the suggested function of scaffold material. But the identification of glial cells as a distinct cell population will be made possible by the development of specific histological stainings such as the silver impregnation method by Golgi (1873). Taking advantage of it, Andriezen (1893a) will distinguish in fact two populations of glial cells that will become known as the protoplasmic and fibrous astrocytes. But the term astrocytes will be coined by von Lenhossek (1893) based on their starlike morphology. Interestingly, Held (1904) proposed that neuroglia rather constitute a syncytium (instead of separate cells), a notion that will be revived later with the discovery of gap junctions between them and will turn out to be important for their metabolic role (Giaume et al., 2010).

Golgi (1886) also made two other important observations. First, he described that each neuroglial cell is in direct contact with a blood vessel through one fine process. With the advent of immunocytochemistry as well as fluorescence and electronic microscopy, not only the confirmation of the presence of astrocytic end-feet on blood vessels was made, but also it was realized that the surface of all capillaries is covered at 99% with these glial elements (Kacem et al., 1998). Moreover, because neuroglial cells are characterized by many fine, dendrite-like, processes and no axons, in contrast to neurons, and that dendrites were assumed to fulfill a nutritive function, by analogy Golgi (1886) hypothesized that neuroglial cells would be dedicated to this role. In accordance with the views of Golgi (1886); Andriezen (1893b) formally proposed that neuroglia (yet not identified as astrocytes) would assume a nutritive function, allowing the transfer of metabolites from the circulation to neurons. He wrote: “*The development of a felted sheath of neuroglia fibers in the ground-substance immediately surrounding the blood vessels of the Brain seems therefore … to allow the free passage of lymph and metabolic products which enter into the fluid and general metabolism of the nerve cells.*” Lugaro (1907) added another aspect by suggesting that neuroglial cells play an essential role in the homeostasis of extracellular milieu, by degrading or taking up substances released by nerve cells for their communication, ensuring a buffer role. Despite these early insights based on histological observations, the metabolic roles of astrocytes were quite controversial at the time and some important scientific figures, like Ramon y Cajal, preferred to consider them rather as sole electrical insulator for nerve cells. More progress on the putative metabolic roles of astrocytes will need to wait for the development of a new field of investigation associated with biochemistry and its methodologies: neurochemistry.

## THE CHEMICAL FACTORY

One major obstacle to determine the functions of astrocytes was the difficulty to study them independently of other cell types within the nervous tissue. In contrast to neurons that are excitable and exhibit complex electrophysiological responses that can be studied individually with fine electrodes *in situ*, astrocytes have more limited electrophysiological features. Astrocytes appeared more interesting from a metabolic point of view but to probe their metabolic characteristics was requiring a distinct approach to be able to study them in isolation. An elegant methodological solution will be proposed by the Swedish scientist Hyden et al. (2000). In the late 1950s, he was able to acutely isolate from the vestibular nucleus of adult animals both neurons and glial cells using thin wires under a stereomicroscope (Hyden, 1959). Using this approach, he was able to determine the metabolic characteristics of each cell type before and after stimulation, using enzymatic measurements. He observed that stimulation led to enhancement of glycolytic capacity in glial cells, and of oxidative capacity in neurons (Hamberger and Hyden, 1963). Based on these results, he postulated the existence of a metabolic cooperation between neurons and glial cells, although the precise nature of the interactions would remain unknown for almost three decades. Indeed, Tsacopoulos et al. (1988) will take advantage of the well-structured organization of the honeybee drone retina to unravel the metabolic role of glial cells. In this preparation, photoreceptor cells are surrounded by a necklace-like set of glial cells easily distinguishable by light microscopy (Tsacopoulos et al., 1988). Using 2-deoxy-D-[5,6-^3^H]glucose (an unmetabolizable analog of glucose) and autoradiography, he could show that all this radioactive tracer was trapped within glial cells (Tsacopoulos et al., 1988). He went on to show that glial cells, that only exhibit glycolytic metabolism, transfer alanine to photoreceptor cells that depend entirely on oxidation of this substrate as source of energy (Tsacopoulos et al., 1994). These data clearly provided the proof of principle that glial cells in general, but eventually astrocytes, do fulfill a metabolic role toward neurons.

With the advent of primary cultures of various brain cell types, it became possible to further explore their individual metabolic properties, including those of astrocytes (McCarthy and de Vellis, 1980). Thus, it was possible to show that astrocytes exhibit a high glycolytic rate with an important production of lactate, as compared to neurons (Walz and Mukerji, 1988). Moreover, they were shown to contain significant levels of glycogen (in contrast to neurons) and this energy reserve could be mobilized by various neuroactive signals including noradrenaline, vasoactive intestinal peptide, adenosine or elevated potassium levels (Magistretti et al., 1981; Sorg and Magistretti, 1991; Hof et al., 1988). Interestingly, the consequence of glycogenolysis in astrocytes was neither an oxidation of the mobilized glycosyl residues nor their release in the extracellular medium. Rather, it was observed that lactate was the end product of glycogenolysis in astrocytes and it was exported outside the cell (Dringen and Hamprecht, 1993; Magistretti et al., 1993). Thus, such a compartmentalization of glycogen, the main energy reserve in the brain, in astrocytes together with the strong production of lactate upon glycogenolysis suggested that these cells may play an important role as energy substrate suppliers for neurons, the main energy consumers of the central nervous system.

Apart from energy supply, astrocytes were also shown to play other important metabolic roles. One of them is glutamate recycling. Indeed, as glutamate is the major excitatory neurotransmitter in the central nervous system, its extracellular concentration needs to be tightly controlled. This is done through a very efficient reuptake system located in astrocytes. High-affinity, sodium-dependent glutamate transporters known as GLT-1 and GLAST were shown to be expressed by astrocytes (Danbolt, 2001). Moreover, the enzyme glutamine synthetase that allows the conversion of glutamate to glutamine was found to be exclusively present in astrocytes (Martinez-Hernandez et al., 1977). Glutamine is then released by astrocytes via a particular aminoacid transporter system, the system N transport (SN1) to be taken up in neurons by a different transport system (system A) before being converted back to glutamate by the enzyme glutaminase (Bröer and Brookes, 2001). It was determined that the great majority (ß80%) of glutamate taken up by astrocytes is converted to glutamine (McKenna et al., 1996). The rest however is oxidized very efficiently and the proportion of oxidized glutamate increases with its concentration. In order to compensate for this cataplerotic use of glutamate, an anaplerotic pathway must exist to replenish the glutamate pool. Astrocytes are able to synthesize glutamate (and glutamine) from glucose via the TCA cycle and aspartate aminotransferase (Pardo et al., 2011). This capacity to maintain glutamate levels for neurotransmission through both the recycling and synthesis of glutamine has been known as the glutamate-glutamine cycle and astrocytes are key elements to support this important neurochemical function.

## GENETIC AND BIOCHEMICAL PROFILING – ESTABLISHING A METABOLIC IDENTITY

It became quite evident that astrocytes appear to be very versatile cells in terms of metabolism. Although they have an important oxidative metabolism especially toward glutamate as described above, they also exhibit a clear aerobic glycolysis capacity. In order to further understand which characteristics are responsible for giving rise to these metabolic responses, both transcriptomic and biochemical investigations have provided some exquisite informations about how astrocytes can combine what appears to be a Pasteur effect with a Warburg effect. Indeed, raising oxygen levels promote oxidative metabolism in astrocytes at the expense of anaerobic glycolysis and lactate production (Pasteur effect). But even in presence of supraphysiological levels of oxygen (e.g., 21% O_2_ in culture conditions), aerobic glycolysis with lactate production was shown to take place in astrocytes (Warburg effect), which can be further enhanced under certain circumstances (e.g., glutamate exposure). The capacity to exhibit both processes may depend on the expression of particular subsets of proteins that need to be specifically identified.

The possibility to explore the level of expression of thousands of genes at once in a selected population of cells using microarrays technology has been applied to acutely isolated, adult astrocytes, thus bypassing the caveats of primary astrocytes in cultures that are essentially obtained from newborn preparations. These studies revealed several interesting points. First of all, they showed that astrocytes express high levels of mitochondrial tricarboxylic acid cycle enzymes, thus confirming the high oxidative capacity of these cells (Lovatt et al., 2007; Cahoy et al., 2008). But at the same time, they showed that astrocytes also strongly express enzymes involved in glycolysis and glycogen metabolism. At the biochemical level, several observations were made that refined the transcriptomic findings. In contrast to neurons, astrocytes maintain high levels of the PFKFB3 protein, a key regulator of glycolysis (Herrero-Mendez et al., 2009). Moreover, the activity of pyruvate dehydrogenase, the key enzyme for the entry into the TCA cycle, is maintained low in astrocytes through its high level of phosphorylation (Itoh et al., 2003; Halim et al., 2010). Finally, it was shown that an important component of the malate-aspartate shuttle in mitochondria, the aspartate glutamate complex Aralar, exhibits a very low expression in astrocytes compared to neurons (Ramos et al., 2003), contributing to a low level of malate-aspartate shuttle activity (Berkich et al., 2007). As a consequence, in order to maintain their high glycolytic rate, astrocytes will prominently convert pyruvate into lactate, thus regenerating the NAD cofactor. Thus, it appears that most of the glucose utilized by astrocytes will not be oxidized within the astrocyte to produce energy. Rather, glucose- or glycogen-derived pyruvate will be converted to lactate and exported, as a consequence of the aforementioned state of key biochemical steps that favor such a metabolic fate. This is further supported by the selective expression of the lactate dehydrogenase B isoform (Bittar et al., 1996; Laughton et al., 2007; O’Brien et al., 2007) and the monocarboxylate transporter MCT4 (Bergersen et al., 2002; Pellerin et al., 2005) by astrocytes which concur with the high glycolytic rate and lactate production capacity of these cells. Although the kinetic characteristics *per se* of these isoforms DO NOT determine metabolite flux direction, their presence is nevertheless indicative of a prevalent metabolic profile, as their properties would be better exploited within such a specific metabolic environment.

## ATTRACTIVE ASTROCYTES – PROBING THE METABOLIC NATURE OF ASTROCYTES WITH MAGNETS

**FIGURE 1:**
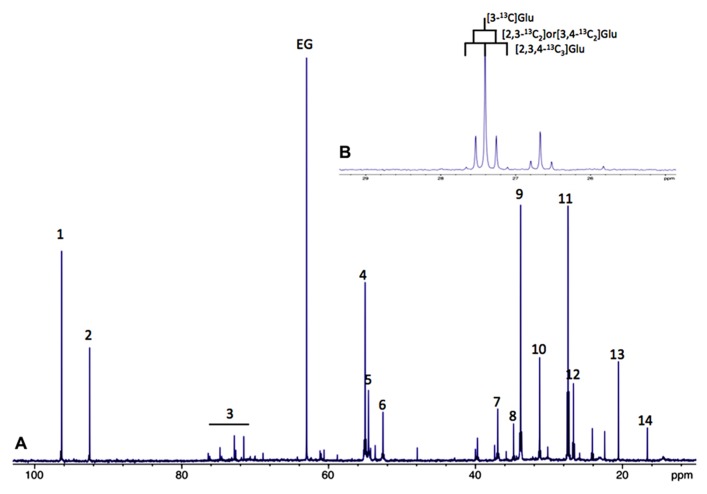
**(A)** Typical ^13^C-NMR spectrum of rat brain perchloric extract, after perfusion with [1-^13^C]glucose. (1) Glucose C1α, (2) glucose C1β, 3: glucose C2, C3, C4, C5 and C6, 4: Glu C2, 5:Gln C2, 6: Asp C2, 7: Asp C3, 8: GABA C2, 9: Glu C4, 10: Gln C4, 11: Glu C3, 12: Gln C3, 13: lactate C3 and 14: Ala C3. **(B)** ^13^C-^13^C coupling figures allow to distinguish between different isotopomers (example on glutamate C3).

A large part of evidence that astrocytes do fulfill a metabolic role towards neurons was achieved by nuclear magnetic resonance (NMR) spectroscopy. ^13^C-NMR spectroscopy in particular is a unique tool to study the metabolism of glucose and metabolic interactions between neurons and astrocytes in the brain. However, sensitivity of the carbon-13 nucleus is low. To overcome these disadvantages, 99%-^13^C enriched substrates, such as [1-^13^C]glucose or [2-^13^C]acetate for example, are used. Added to the cell culture medium, or intravenously injected, this magnetic active isotope will permit analyzing cellular metabolism over time using ^13^C-NMR spectroscopy. Indeed, all ^13^C -labeled metabolites derived from the ^13^C-labeled precursor will be detected on a single ^13^C-NMR spectrum; each carbon will respectively give a signal (peak) at a different place on the NMR scale depending on their position within every metabolite. (**Figure 1A**).

Moreover, it is also possible to detect on the same spectrum if one carbon 13 is linked to an unlabeled carbon 12 or to another carbon 13. In this latter case, homonuclear spin coupling patterns will appear (**Figure 1B**). For example, a ^13^C with one ^13^C neighbor will lead to a doublet (instead of a singlet if linked to a carbon 12); with two ^13^C neighbors, the peak will become a triplet and so on, the rule being *n*+1 peaks where n equals the number of ^13^C neighbors. ^13^C-NMR spectroscopy is therefore a powerful technique which can be applied *in vitro*, *ex vivo* and *in vivo* to follow up labeled carbons in metabolites and examine their fate through different metabolic pathways.

### *IN VITRO* STUDIES

**FIGURE 2:**
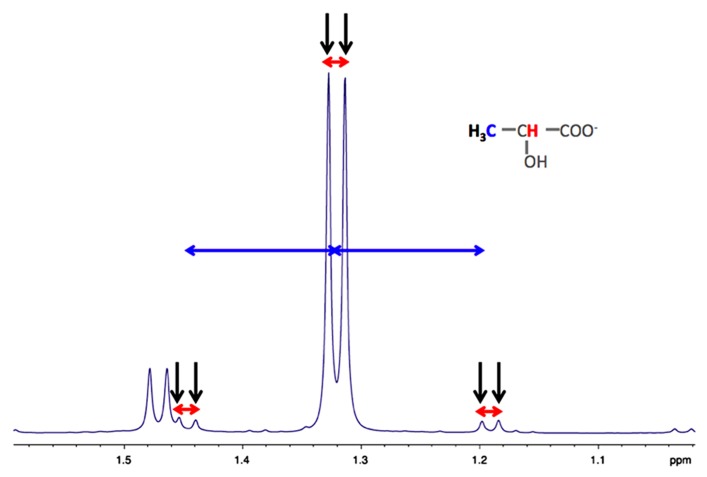
**Typical high resolution at the magic angle spinning (HRMAS) ^1^H-NMR spectrum of rat brain biopsy after [3-^13^C]lactate perfusion**. Protons of the methyl group of lactate are detected (black arrows), centered at 1.32 ppm. The doublet is coming from the homonuclear spin coupling (J_H-H_ = 7 Hz, red arrows). When a ^13^C is located on lactate carbon 3, then a doublet of doublet is appearing (^13^C satellites of H_3_ lactate), due to the heteronuclear spin coupling (J_H-C_ = 128 Hz, horizontal blue arrows).

As indicated in the first part of this review, astrocytes exhibit a clear aerobic glycolysis. NMR spectroscopy is particularly suitable to estimate the rate of glycolysis in astrocytes, by measuring the rate of lactate formation. ^1^H-NMR spectroscopy allows detecting on the same spectrum, the ^13^C-labeled lactate synthetized from glycolysis of the administered ^13^C-labeled glucose, and also, the unlabeled lactate coming from unlabeled precursors. Indeed, as shown in **Figure 2**, on carbon 3 of the unlabeled lactate, the protons of the methyl group will give a doublet at 1.32 ppm, rising from their homonuclear coupling (^1^H/^1^H) with the neighbor ^1^H linked to carbon 2 (**Figure 2**, in red). On the other hand, the [3-^13^C] lactate will lead to two doublets, at 1.21 and 1.43 ppm due to the heteronuclear coupling (^1^H/^13^C, different coupling value *J* = 128 Hz).

The first experiments using NMR spectroscopy on brain cell cultures were conducted* in vitro* in the early 90’s. The metabolism of [1-^13^C]glucose by astrocytes, neurons and mixed astroglial/neuronal cultures derived from the striatum of fetal rats was studied by Leo et al. (1993). Interestingly, they found that neuronal cultures consumed glucose much slower than the astrocytic or the mixed cultures. In the study of Martin et al. (1993) they investigated the metabolism of [1-^13^C]glucose in rat cerebellum astrocytes and granule neurons. Results showed that the ^13^C-specific enrichment of lactate C3 (% of ^13^C incorporated into the carbon position 3 of lactate from the precursor [1-^13^C]glucose enriched at 99%) was higher in astrocytes compared to neurons, demonstrating that astrocytes were more glycolytic than neurons. Moreover, although acetylCoA C2 and lactate C3 had very similar enrichments in granule cells, acetylCoA C2 enrichment in astrocytes was 60% lower than that of lactate C3. These data indicate that the labeling at the pyruvate node was mainly directed toward the TCA cycle in neurons, which was not the case for astrocytes. This glycolytic feature of astrocytes was also demonstrated on mouse primary cultures (Sonnewald et al., 1993). When astrocytes were incubated with [1-^13^C]glucose, lactate C3 was found to be enriched at 30%. Since the maximum theoretical labeling value is 50% (as one [1-^13^C]glucose molecule gives rise to two pyruvate, and thus to two lactate molecules, one labeled and one unlabeled), we can calculate that 60% of glucose was converted into lactate. Based on the rates of glucose consumption and lactate production, this percentage was found even higher in another study (ranging between 67 and 84%), also performed on primary cultures of mouse astrocytes (Teixeira et al., 2008). A more recent study, combining NMR and metabolic flux analysis, confirmed that astrocytes showed a high glycolytic flux, converting most of the glucose to lactate (Amaral et al., 2011). This particular astrocytic metabolic characteristic was also observed even if a high concentration of lactate was present (Alves et al., 1995). In this latter study, astrocytes were incubated in a medium containing 6 mM of [1-^13^C]glucose and 10 mM of lactate. After 6 h, [3-^13^C]lactate was detected in the medium and its specific enrichment was 6.5%. Combining this value with the total amount of lactate present at *t* = 6 h (162 mmol/mg prot) and the rate of glucose consumption (136 mmol/mg prot in 6h), we can estimate that 10.5 mmol/mg prot of [3-^13^C]lactate were produced in 6h and that around 15% (10.5 × 2/136) of glucose was converted into lactate and exported out of the cell. This experiment reinforces the glycolytic nature of astrocytes even if high concentrations of lactate are present in the medium.

Beside [1-^13^C]glucose, other ^13^C-labeled substrates were tested. The fate of [3-^13^C]alanine was followed and compared between primary cultures of astrocytes, neurons and co-cultures (Zwingmann et al., 2000). In astrocytes, 90% of the [3-^13^C]alanine was converted into [3-^13^C]lactate, whereas only a 12.5%-conversion was measured in neurons. The increased glycolytic activity in astrocytes induced by the uptake of alanine was suggested to contribute to the synthesis of releasable lactate. This alanine-lactate shuttle might constitute a way to transfer nitrogen from neuron to astrocytes (Zwingmann et al., 2001; Bak et al., 2005), which may promote, in return, the glutamate-glutamine cycle between these two cell types.

The fate and metabolism of ^13^C-labeled lactate was also explored. Primary cultures of mouse cortical astrocytes were incubated during 4 h in a medium without glucose and containing 1 mM [U-^13^C_3_]lactate (Waagepetersen et al., 1998a). The incorporation of ^13^C into glutamate was only 50% of the corresponding one observed in cultured neocortical neurons cultured under the same conditions (Waagepetersen et al., 1998b). In parallel to the high glycolytic activity and lactate production in astrocytes, this result suggests that lactate is predominantly employed as an oxidative substrate in neurons. From these two studies, we can also compare the ^13^C-NMR spectra of neurons incubated with either 1 mM [U-^13^C_3_]lactate (Waagepetersen et al., 1998b) or 1 mM [U-^13^C_6_]glucose (Waagepetersen et al., 1998a); we can clearly observe that more carbon-13 was incorporated into glutamate in the lactate-labeled condition. To determine which is the preferential neuronal substrate, a competition between glucose and lactate was performed (Bouzier-Sore et al., 2003). Both substrates were added to the culture medium, but alternatively labeled ([1-^13^C]glucose + lactate or glucose + [3-^13^C]lactate). When glucose and lactate concentrations were equal (5.5 mM), results clearly indicated that neurons in the presence of both substrates preferentially use lactate as their main oxidative substrate. The same result was found under physiological concentrations of glucose and lactate (1.1 mM; Bouzier-Sore et al., 2006). Using a mathematical model, the relative contribution of exogenous glucose and lactate to neuronal oxidative metabolism was measured to be 25% for glucose and 75% for lactate.

Metabolism on brain slices can also be explored by NMR spectroscopy (Badar-Goffer et al., 1992). Guinea-pig cerebral-cortical slices were incubated with either [1-^13^C]glucose or [2-^13^C]acetate, a more specific glial substrate (Waniewski and Martin, 1998), under resting or depolarization conditions. When [1-^13^C]glucose was the labeled substrate, an intense and much higher lactate resonance was observed on the ^13^C-NMR spectrum during activation compared to resting conditions. Lactate C3 specific enrichment was 45% (close to the theoretical 50% value). Moreover, under depolarization, it was evidenced that glucose metabolism in glia was selectively stimulated: a significant increase in ^13^C-incorporation was occurring into metabolites of the glial pool.

Altogether, these *in vitro* results obtained on separate astrocytic and neuronal cultures support the idea that astrocytes exhibit a clear aerobic glycolysis and produce lactate. It can then be used as a supplementary fuel by neurons since lactate appears to be a more efficient oxidative substrate for them compared to astrocytes.

### *EX VIVO *AND* IN VIVO* STUDIES

Glial-neuronal metabolic interactions can be studied using ^13^C-labeled substrates and high resolution or *in vivo* ^13^C-NMR techniques. Compared to *in vitro* studies, *ex vivo* or *in vivo* experiments are more complicated to interpret since all metabolites from the different cell types are present on the same spectrum. However, it is possible to distinguish astrocytic from neuronal metabolism since a metabolic and enzymatic compartmentalization exists between neurons and astrocytes. Indeed, the existence of two distinct cerebral pools of glutamate was first determined; a small one (around 10%) attributed to the astrocyte compartment and a large neuronal one (Berl et al., 1962; Van den Berg et al., 1969). Thereafter, it was shown that glutamine synthetase and glutaminase were exclusively glial (Martinez-Hernandez et al., 1977) and mainly neuronal (Patel et al., 1982), respectively. The key outcome of this enzymatic compartmentalization is the glutamate-glutamine cycle between neurons and astrocytes. Moreover, since glutamate is in rapid equilibrium with the TCA cycle intermediate α-ketoglutarate, the neuronal TCA cycle flux can be estimated from the kinetics of ^13^C enrichment of total cerebral glutamate (Fitzpatrick et al., 1990; Mason et al., 1992, 1995; Sibson et al., 1998, 2001). Since glutamine synthetase is exclusively located in astrocytes, glutamine will reflect the astrocytic compartment. Moreover, pyruvate carboxylase (PC) was found to be also only in astrocytes (Yu et al., 1983; Shank et al., 1985). The presence of this enzyme will lead to a different fate of the ^13^C compared to neurons and to a higher incorporation of the ^13^C into the carbon position 2 compared to carbon position 3 in glutamine. Such imbalance cannot be evidenced for glutamate, which reflects the neuronal compartment, where PC activity is not present. Astrocytic metabolism can also be distinguished from the neuronal one using ^13^C-labeled acetate since this substrate is only transported to glial cells (Waniewski and Martin, 1998). This substrate enters the TCA cycle directly at the citrate level, bypassing thus the PC and PDH steps. Using all these tools, neuronal and astrocytic TCA cycle rates can be estimated either from rat brain extracts (Kunnecke et al., 1993; Preece and Cerdan, 1996) or directly *in vivo* after infusion of ^13^C-labeled substrates (; Sibson et al., 1997; Shen et al., 1999; Lebon et al., 2002). Glial TCA cycle rate was found to be 0.4 and 0.14 mmol/min/g, in rat brain extracts and human brain, respectively, whereas neuronal TCA cycle rate was 1 mmol/min/g in rat brain extracts and ranging from 0.6 to 1.6 mmol/min/g in *in vivo* experiments. This demonstrated a higher neuronal oxidative metabolism, compared to glia’s one (Rodrigues et al., 2012).

The exclusive presence of the PC enzyme in astrocytes was used to analyze the fate of [3-^13^C]lactate in rat brain extracts (Bouzier et al., 2000; Hassel and Brathe, 2000) and *in vivo* in humans (Boumezbeur et al., 2010). When rats received an intravenous infusion of [3-^13^C]lactate, the analysis of the ^13^C-NMR spectrum of the brain extracts indicated that no imbalance between glutamine carbon 2 and carbon 3 could be evidenced. Such data indicate that there was no entry of ^13^C into the astrocytic TCA cycle via the PC pathway, and therefore that [3-^13^C]lactate was metabolized in a PC-deprived compartment, i.e., neurons. This lactate consumption has also been confirmed to be more neuronal specific *in vivo* in humans (Boumezbeur et al., 2010). Interestingly, a correlation was found between the use of endogenously synthetized lactate and level of activity (Serres et al., 2004). Finally, it was recently shown that during rat brain activation (whisker stimulation) there was an average 2.4-fold increase in lactate content in the activated area. Furthermore, this increase was arising from newly synthetized lactate during brain activation from blood ^13^C-labeled glucose (Sampol et al., 2013).

## SEEING IS BELIEVING – THE CONTRIBUTION OF FLUORESCENCE IMAGING

Despite the power of NMR to obtain metabolic information, this technique is unable to give an answer at the cellular level. The need to visualize metabolic responses from individual cells especially *in situ* became essential. Different optical approaches have been exploited to attain this goal. Another advantage of optical methods is that they allow the characterization of fast metabolic events, which can be applied on preparations with mixed populations of cells. This could be particularly important since the metabolic maturation of astrocytes might depend on signals from other cell types (Brix et al., 2012). A first method is based on the intrinsic fluorescence produced by the metabolic co-factor NADH. Major changes in intracellular NADH fluorescence have been attributed to alterations in mitochondrial activity (Mayevsky and Rogatsky, 2007). However, a cytosolic NADH fluorescence signal can be evidenced in astrocytes and associated with an enhancement of glycolysis (Kasischke et al., 2004; Requardt et al., 2010). These characteristics will be exploited in combination with two-photon microscopy to study the metabolic responses of brain cells and specifically in astrocytes upon stimulation both *ex vivo* (in slices) and *in vivo*.

It was demonstrated in hippocampal brain slices that electrical stimulation produced a biphasic signal of intrinsic NADH fluorescence (Kasischke et al., 2004; Brennan et al., 2006). An early dip in NADH fluorescence was observed followed by a delayed increase of the signal. The early decrease in NADH signal was associated with enhanced oxidative metabolism in neurons (Kasischke et al., 2004; Brennan et al., 2006), most likely due to enhanced lactate utilization (Galeffi et al., 2007). Although the enhancement in NADH signal subsequently taking place could be largely due also to oxidative metabolism in neurons (Brennan et al., 2006), a delayed increase of the fluorescence signal originating from the cytosol of astrocytes also occurred in parallel (Kasischke et al., 2004). Such a response in astrocytes seems to be caused by an enhancement of glycolysis in these cells, as revealed both in cultured astrocytes and in cortical brain slices stimulated with dopamine (Requardt et al., 2010). A stunning confirmation of this sequence of events was provided *in vivo* in the cerebellum using flavoprotein autofluorescence imaging (Reinert et al., 2011). The first part of the response observed, called the on-beam light phase, could be attributed essentially to activation of oxidative metabolism in neurons. Of note, lactate oxidation in neurons seems to participate to the on-beam light phase signal. The second part identified as the on-beam dark phase appears to be dependent, at least in part, on activation of glutamate transporters in glia and could be caused by the reduction of flavoproteins via an increase in glycolysis, although the origin of the dark phase signal cannot be attributed specifically either to glia or neurons. It was also suggested (but not demonstrated) that elevated extracellular potassium could be another factor contributing to the on-beam dark phase via its stimulation of glial glycolysis.

An important question to be addressed was the degree of glucose utilization by both neurons and astrocytes. Indeed, based on the estimated energy expenditures of each cell type, it is predicted that the majority (>70%) of glucose consumption should occur in neurons while the remaining (<30%) should take place in glial cells, assuming that glucose is entirely oxidized (Attwell and Laughlin, 2001; Pellerin and Magistretti, 2003). Two approaches have been developed in order to evaluate glucose uptake and utilization by each cell type. First of all, FRET nanosensors can be used to measure the intracellular concentration of glucose and estimate glycolytic rates in specific cells, including astrocytes and neurons (Bittner et al., 2010). The use of fluorescent glucose analogs such as 2- and 6-NBDG can also be used to evaluate the relative glucose uptake and utilization by neurons vs. glia. Thus, it was shown first in cerebellar slices that most glucose uptake and utilization takes place in Bergmann glia and not in Purkinje neurons (Barros et al., 2009). More recently, a follow-up study was performed in both cerebellar and hippocampal slices in which glucose transport and metabolism was found to be faster in Bergmann glia and astrocytes than in neurons (Jakoby et al., 2013). The results led to the conclusion that preferential glucose transport and metabolism takes place in glia. Interestingly, it was demonstrated that 6-NBDG, the glucose analog used to estimate glucose transport, largely underestimates glucose transport in astrocytes compared to neurons. Thus, it is clear that the rate of glucose transport and utilization is largely superior in astrocytes vs. neurons. Such a conclusion has important consequences. As stated above, if glucose is the sole energy substrate used by brain cells, it is expected that glucose transport and utilization should be proportional to the cell energy needs. Clearly, this is not the case. The most likely explanation to resolve this paradox is to admit that astrocytes convert a substantial amount of the glucose they use into lactate. Then, the lactate released by astrocytes can be used by neurons as an additional oxidative substrate to satisfy their large energy needs (Pellerin and Magistretti, 2003).

Data above provided indications about the glycolytic capacity of astrocytes *in vitro* and *ex vivo* (in slices) under resting condition. It was necessary to obtain further insight *in vivo* under both resting and activated conditions. Two-photon microscopy imaging was performed over the rat somatosensory cortex upon infusion of 6-NBDG (Chuquet et al., 2010). At rest, the amount of 6-NBDG accumulating in astrocytes and neurons was equivalent. But based on the higher affinity of 6-NBDG for the glucose transporter expressed by neurons (GLUT3) compared to the one found on astrocytes (GLUT1), it seems that already at rest, the largest proportion of glucose is taken up by astrocytes. Upon whisker stimulation, most of the increased 6-NBDG accumulation took place in astrocytes. These results provide a strong evidence that astrocytes are the major site of glucose uptake and utilization in the brain. They also respond to neuronal activation by enhancing their glucose uptake and utilization. The mechanisms explaining such a specific metabolic response of astrocytes have been clarified over the years by the use of cell culture preparations.

First of all, using isotopic methods, glutamate had been clearly shown to cause an enhancement of glucose utilization in cultured astrocytes by a mechanism involving its uptake and an activation of the Na^+^/K^+^ ATPase (Pellerin and Magistretti, 1994; Takahashi et al., 1995). Such an effect of glutamate on astrocytes was confirmed *in vivo* (Voutsinos-Porche et al., 2003). In contrast, potassium was found to have either a small (Brookes and Yarowsky, 1985) or no effect (Takahashi et al., 1995) on glucose utilization in cultured astrocytes. With the advent of optical methods allowing measurements with high temporal resolution, a better characterization of the role of each substance could be performed. Indeed, taking advantage of a FRET glucose nanosensor, the group of Felipe Barros was able to show that while potassium caused a rapid but transient enhancement in the glycolytic rate (explaining why it was overlooked in isotopic studies), glutamate had a delayed but long-lasting effect (Bittner et al., 2011). Moreover, using the same approach, the same group was able to demonstrate that the glycolytic action of potassium in astrocytes requires the implication of the Na^+^ /HCO_3_^-^ cotransporter NBCe1, while the Na^+^/K^+^ ATPase only plays a permissive role in this case (Ruminot et al., 2011). Interestingly, while they could also observe using the fluorescent glucose analogs 2- and 6-NBDG the enhancing effect of glutamate on glucose transport in cultured astrocytes (Loaiza et al., 2003), they found just the opposite in cultured neurons (Porras et al., 2004). These data are consistent with the concept that while neuronal activity triggers an enhancement of glucose uptake and glycolysis in astrocytes, it rather prevents glucose utilization in neurons under physiological conditions. As mentioned earlier, in contrast to astrocytes, neurons normally expressed low levels of the key regulator of glycolysis PFKFB3 (Herrero-Mendez et al., 2009). It is only under excitotoxic conditions leading to overstimulation of NMDA receptors that neuronal glycolysis can be activated (Rodriguez-Rodriguez et al., 2012, 2013) and neuronal glucose utilization be increased (Bak et al.; 2009), but this condition leads to neuronal cell death (Rodriguez-Rodriguez et al., 2012, 2013).

Most results converge toward the idea that astrocytes are the main brain cell type not only consuming glucose but also exhibiting glycolytic responses upon neuronal activation. In contrast, neurons appear to be highly oxidative cells that would prefer to oxidize lactate rather than produce it from glucose. A key question that arises is what are the key metabolic features that determine the apparently different (but complementary) metabolic phenotypes of astrocytes and neurons. Possible hints are emerging from modeling studies.

## CALCULATE ME AN ASTROCYTE – MATHEMATICAL MODELING

Different modeling efforts have attempted to capture the role that astrocytes might play as suppliers of energy substrates for neurons along with their other metabolic functions. A first approach was proposed by developing a model of compartmentalized brain energy metabolism whereby astrocytes and neurons have been dissociated and assumed to exhibit slightly different metabolic features, based on the experimental data available (Aubert and Costalat, 2005). In such case, it was found that despite assumptions highly unfavorable to a popular concept of energy substrate supply between brain cells known as ANLS (for astrocyte-neuron lactate shuttle; see Pellerin and Magistretti, 2012), neuronal activation led to a robust lactate flux from astrocytes to neurons that can be either continuous or phasic, depending of the degree of neuron vs. astrocyte activation. This model was pushed one step further to address the question of brain lactate kinetics. Taking this time into account the distribution and kinetics of monocarboxylate transporters involved in lactate transport as well as the variations in extracellular lactate levels, it could be concluded that neurons represent the most likely compartment where lactate is consumed while astrocytes would be a plausible source (Aubert et al., 2005). Such a cellular compartmentalization of brain energy metabolism was supported by another modeling approach based rather on brain glucose and oxygen utilization (Jolivet et al., 2009). The authors concluded that glycolysis must take place in large part in astrocytes (while oxidative metabolism would predominate in neurons) and that glucose-derived metabolites must be transferred from glial cells to neurons. Independently, other authors have shown with their modeling approach that lactate shuttling from astrocytes to neurons could be advantageous for neurons, both under normoxia and hypoxia, further extending the validity of the concept to pathological situations (Genc et al., 2011). They also emphasized the fact that astrocytes and neurons might switch between a more classical, glucose alone-based mode of metabolism to a metabolic interaction mode, depending of the situation.

However, another contrasting view has been proposed following a different series of modeling analyses. It has been argued, based on a distinct set of data, that there is probably very little shuttling of lactate from astrocytes to neurons (DiNuzzo et al., 2010a). If anything, it was even proposed that it is rather the neurons that export lactate while astrocytes would oxidize it (Mangia et al., 2009). Similarly, it was proposed that glycogenolysis, a process known to occur only in astrocytes, would serve the purpose of funneling glucose to neurons instead of shuttling lactate to them (DiNuzzo et al., 2010b), in sharp contrast to the well established data showing that lactate rather than glucose is released by astrocytes following glycogenolysis as indicated above (Dringen and Hamprecht, 1993; Magistretti et al., 1993). This controversy gave rise to a heated debate, each side providing arguments to dismiss the conclusions of the other (Jolivet et al., 2010; Mangia et al., 2011; Pellerin and Magistretti, 2012). Some authors have attempted to reconcile the two points of view by applying a more probabilistic approach of modeling (Somersalo et al., 2012). After a rigorous and thorough analysis of each model, their conclusion is that there is such variability in the system that each one might capture only one part of the reality, advocating for stochastic models rather than deterministic ones.

**FIGURE 3:**
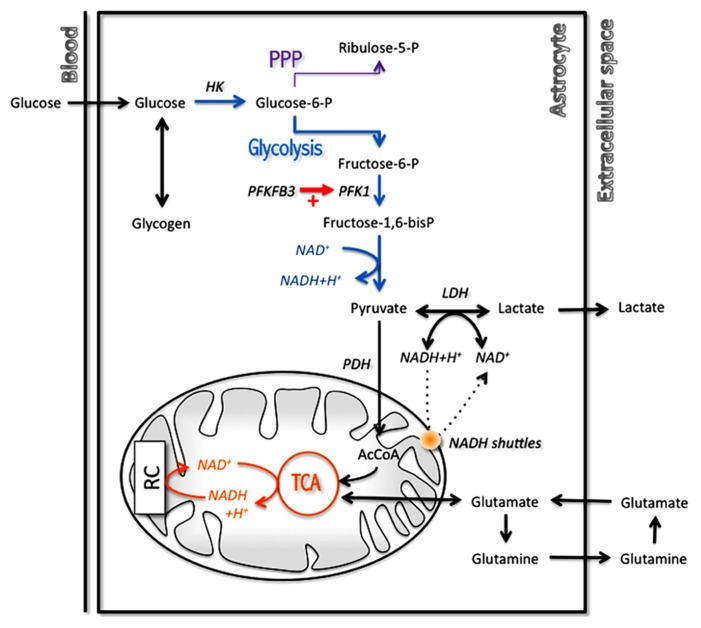
**Main metabolic pathways implicated in energy production in astrocytes with a prominent role for glycolysis.** In order to regenerate cytosolic NAD^+^ levels and maintain glycolytic rate, astrocytes have two options: transfer cytosolic NADH in mitochondria through specific mitochondrial NADH shuttles or convert pyruvate into lactate in the cytosol. AcCoA, acetylCoenzyme A; HK, hexokinase; LDH, lactate dehydrogenase; PDH, pyruvate dehydrogenase; PFK1, phosphofructokinase 1; PFKFB3, 6-phosphofructo-2-kinase/fructose-2,6-biphosphatase 3; PPP, pentose phosphate pathway; RC, respiratory chain; TCA, tricarboxylic acid cycle.

Nevertheless, there are still some interesting points that have been highlighted, confirming for example experimental data. Thus, it was confirmed that astrocytes have a TCA cycle rate several orders of magnitude lower than neurons (Occhipinti et al., 2007), as was determined previously both *in vitro* (Bouzier-Sore et al., 2006) and *in vivo* (Tyson et al., 2003). This is not to say that they are devoid of oxidative activity as it was previously demonstrated (Wyss et al., 2009) but at least it does not compare to the degree observed in neurons. Moreover, a recent modeling study has tackled the critical question of which biochemical steps determine whether a cell is rather oxidative (thus oxidizing both glucose and lactate) or exhibit some glycolytic features (by exporting rather than consuming lactate; Neves et al., 2012). This work has revealed that the flux through the pyruvate dehydrogenase-catalyzed reaction as well as the mitochondrial NADH shuttling rate are essential in determining the preference for oxidation rather than for export of lactate (**Figure 3**). Varying the importance of these two reactions by a modest value allowed to observe a switch in metabolic phenotype. Interestingly, it was observed that astrocytes in general exhibit characteristics for these two steps (i.e., low pyruvate dehydrogenase and mitochondrial NADH shuttling activities; see above Itoh et al., 2003; Ramos et al., 2003; Halim et al., 2010) that are consistent with the prominence of aerobic glycolysis in this cell type, in contrast to neurons that are in most cases essentially oxidative in nature. In addition to these features that determine the overall metabolic profile in resting state, there is also other mechanisms that come into play in a transient manner during activated states and further reinforce these characteristics. This is the case in astrocytes for which it was shown that in parallel with glutamate uptake that follows glutamatergic activity, an intracellular acidification takes place that spreads over mitochondria (Azarias et al., 2011). As a consequence, the cytosol-to-mitochondrial matrix pH gradient is abrogated, reducing oxidative metabolism in this cell type. Such a mechanism would favor glycolysis in astrocytes and spare oxygen for its use by neuronal oxidative metabolism. Modeling of these metabolic transients might provide us with further insights about the dynamic aspect of these adaptive metabolic characteristics of astrocytes.

## THE INDISPENSABLE ASTROCYTE – IMPLICATIONS IN VARIOUS BRAIN FUNCTIONS

The putative roles related to the metabolic characteristics of astrocytes are just beginning to be explored but there is already a number of conditions for which their importance as started to be highlighted. Apart from the well-characterized role as lactate supplier for active neurons (Pellerin and Magistretti, 2012), the possibility that lactate produced by astrocytes could be a coupling factor to link neuronal activity to increased blood flow has been revealed (Gordon et al., 2008). Thus, it appears that astrocytes, through their metabolic response, represent key elements in both the neurovascular and the neurometabolic coupling, two mechanisms at the basis of functional brain imaging (Bonvento et al., 2002).

It was previously suggested that enhanced glycolysis and glycogenolysis in astrocytes were essential for the formation of memory in neonate chicks (O’Dowd et al., 1994a, 1994b). More recently, it was shown that lactate, produced by astrocytes by those two metabolic pathways and transferred to neurons via monocarboxylate transporters, is essential for memory formation in rodents (Newman et al., 2011; Suzuki et al., 2011). Other central functions have also been shown to be dependent on proper astrocyte-neuron metabolic interactions. Thus, it was shown that orexin neurons, that play a key role in arousal, are sensitive to astrocyte-derived lactate and modify their firing activity (Parsons and Hirasawa, 2010). Sleep is another centrally controlled condition that might be regulated by the energetic responses of astrocytes (Scharf et al., 2008). Indeed, the metabolism of glycogen, which is essentially present in astrocytes, has long been associated with sleep/wake cycle regulation (Benington and Heller, 1995).

Different peripheral functions controlled by the central nervous system seem also to be regulated via metabolic responses of astrocytes. This is the case of glucose sensing that is regulated at the level of the hypothalamus. It was shown that regulation of blood glucose depends on the conversion of glucose into lactate, presumably in astrocytes, and lactate metabolism in neurons (Lam et al., 2005). Similarly, respiration control was shown to depend on proper metabolic interactions between astrocytes and neurons (Erlichman et al., 2008). Thus, it was demonstrated that in the retrotrapezoid nucleus, astrocytes participate to the medullary central chemosensory stimulus by providing lactate to neurons. Sodium homeostasis is another essential function for the organism and it is regulated centrally at the level of the subfornical organ. It was shown that elevation of sodium level in body fluids is detected by astrocytes and ependymal cells in the subfornical organ and transmitted to neurons via a lactate signal, allowing to regulate their activity, and set in motion the appropriate adaptative responses (Shimizu et al., 2007).

It is likely that if so many important brain functions depend on appropriate astrocyte-neuron metabolic interactions, some pathological situations might be caused by a dysfunction or failure in this process at one level or another. It could be the case for Alzheimer’s disease as it was recently demonstrated (Allaman et al., 2010). Indeed, it was found that β-amyloid aggregates alter the metabolic phenotype of astrocytes and in return, affect neuronal viability. As this case illustrates, astrocytes and their metabolic properties might represent an interesting therapeutic target in various neurological diseases. Some studies have already shown the putative neuroprotective impact of modifying the intrinsic metabolic characteristics of astrocytes, either by exposing them to specific trophic factors (Escartin et al., 2007) or by overexpression of intrinsic metabolic components (Bliss et al., 2004).

Based on what we have seen so far about their metabolic capacities, it is clear that astrocytes have not finished to surprise and fascinate us. And this is precisely what we expect from the stars of the brain.

## AUTHOR CONTRIBUTIONS

Anne-Karine Bouzier-Sore and Luc Pellerin conceived the manuscript, wrote and corrected the text.

## Conflict of Interest Statement

The authors declare that the research was conducted in the absence of any commercial or financial relationships that could be construed as a potential conflict of interest.
